# Dystonia and Paroxysmal Dyskinesias: Under-Recognized Movement Disorders in Domestic Animals? A Comparison with Human Dystonia/Paroxysmal Dyskinesias

**DOI:** 10.3389/fvets.2015.00065

**Published:** 2015-11-30

**Authors:** Angelika Richter, Melanie Hamann, Jörg Wissel, Holger A. Volk

**Affiliations:** ^1^Faculty of Veterinary Medicine, Institute of Pharmacology, Pharmacy and Toxicology, University of Leipzig, Leipzig, Germany; ^2^Department of Veterinary Medicine, Institute of Pharmacology and Toxicology, Free University Berlin, Berlin, Germany; ^3^Department of Neurological Rehabilitation and Physical Therapy, Vivantes Hospital Spandau and Humboldt Hospital, Berlin, Germany; ^4^Department of Neurology, Vivantes Hospital Spandau and Humboldt Hospital, Berlin, Germany; ^5^Clinical Science and Services, The Royal Veterinary College, Hatfield, UK

**Keywords:** dystonia, dyskinesia, hyperkinetic movement disorder, veterinary, domestic animals

## Abstract

Dystonia is defined as a neurological syndrome characterized by involuntary sustained or intermittent muscle contractions causing twisting, often repetitive movements, and postures. Paroxysmal dyskinesias are episodic movement disorders encompassing dystonia, chorea, athetosis, and ballism in conscious individuals. Several decades of research have enhanced the understanding of the etiology of human dystonia and dyskinesias that are associated with dystonia, but the pathophysiology remains largely unknown. The spontaneous occurrence of hereditary dystonia and paroxysmal dyskinesia is well documented in rodents used as animal models in basic dystonia research. Several hyperkinetic movement disorders, described in dogs, horses and cattle, show similarities to these human movement disorders. Although dystonia is regarded as the third most common movement disorder in humans, it is often misdiagnosed because of the heterogeneity of etiology and clinical presentation. Since these conditions are poorly known in veterinary practice, their prevalence may be underestimated in veterinary medicine. In order to attract attention to these movement disorders, i.e., dystonia and paroxysmal dyskinesias associated with dystonia, and to enhance interest in translational research, this review gives a brief overview of the current literature regarding dystonia/paroxysmal dyskinesia in humans and summarizes similar hereditary movement disorders reported in domestic animals.

## Introduction

Dystonia is characterized by sustained or intermittent muscle contractions causing abnormal, often repetitive, movements, postures, or both. Dystonic movements, which are typically patterned, twisting and sometimes tremulous, can be initiated or worsened by voluntary action (1). Dystonia is the third most common movement disorder in humans and comprises a large number of types, currently classified by (1) clinical characteristics (age at onset, body distribution, temporal pattern and associated features) and (2) etiology (nervous system pathology, inherited or acquired), as summarized in Table 1. Although the term dyskinesia includes a wide range of abnormal, involuntary movements that can be associated with many neurological disorders, it is currently mainly used for paroxysmal dyskinesias (Table 2) and drug-induced dyskinesias in human neurology (2, 3). Paroxysmal dyskinesias are characterized by self-limiting episodes, usually induced by stress or sudden movements (4). These episodic disorders include dystonia and also other abnormal involuntary, non-stereotypical movements, such as chorea (rapid, distal dance-like movements), athetosis (slow writing movements), and ballism (rapid, more violent, predominantly involving proximal muscle groups). The term “hyperkinesia” includes these syndromes as well as any unwanted excess movements (5). Dystonia can be the dominant feature in paroxysmal dyskinesias, termed “paroxysmal dystonia” in Table 1 (1). Diagnosing dystonia has been described as difficult, because of variability in clinical presentation, the wide etiological spectrum, and possible coexistence with other movement disorders (6). It has been estimated that about 3 million people suffer from dystonia worldwide (7, 8).

**Table 1:** **Classification of dystonia (1)**.

**(A) CLINICAL CHARACTERISTICS OF DYSTONIA**
**Age of onset** (infancy, childhood, adolescence, and early/late adulthood)
**Body distribution**
Focal (only one body region is affected. e.g., blepharospasm)
Segmental (two or more contiguous body regions are affected)
Multifocal (two non-contiguous or more – contiguous or not – body regions are involved)
Hemidystonia (more body regions restricted to one body side are involved)
Generalized (involvement of the trunk and at least two other sites, including leg or not)
**Temporal pattern**
Course of disease (static or progressive)
Variability
Persistent (appears to approximately the same extent throughout the day)
Diurnal (recognizable circadian variations in occurrence, severity and phenomenology)
Action-specific (occurs only during a particular activity or task)
Paroxysmal (sudden self-limited episodes of dystonia usually induced by a trigger)
**Associated features**
Isolated dystonia (dystonia is the only sign, with the exception of tremor)
Combined with other movement disorders (e.g., myoclonus)
Associated with other neurological symptoms (e.g., cognitive decline) or systemic manifestations (e.g., Wilson disease)
**(B) ETIOLOGY**
**Nervous system pathology**
Evidence of degeneration
Evidence of structural (often static) lesions
No evidence of degeneration or structural lesion
**Inherited or acquired**
Inherited (autosomal dominant, autosomal recessive, X-linked recessive, and mitochondrial)
Acquired (brain injury, infection, drug, toxic, vascular, neoplastic, and psychogenic)
Idiopathic (sporadic and familial)

**Table 2:** **Types of paroxysmal dyskinesias (4)**.

Term	Duration	Frequency	Trigger	Response to antiepileptic drugs
Paroxysmal kinesigenic dyskinesia (PKD)	Typically <2 min	Up to 100 per day	Sudden movements	Often effective
Paroxysmal non-kinesigenic dyskinesia (PNKD)	5 min to 4 h	None for months to few per day	Excitement, stress, fatigue, alcohol, and caffeine	Usually not effective
Paroxysmal exertion induced dyskinesia (PED)	5–30 min	One per month to daily	Prolonged muscle exertion	Usually not effective
Paroxysmal hypnogenic dyskinesia (PHD); type of frontal lobe epilepsy	30–45 s	5 times a year to five times a night	During non-REM sleep	Sometimes effective
Paroxysmal torticollis of infancy	Hours to days	Every 2 or 3 weeks	CHanges in posture	Spontaneous disappearance before the age of 5 years

In laboratory animals, dystonic phenotypes caused by spontaneous mutations are well-known, as described in previous reviews as animal models of dystonia (9–12). It is, therefore, unlikely that this movement disorder is restricted to human patients. In domestic animals, it is probably often unrecognized and becomes a reason for euthanasia in severe and intractable cases. Several case reports of “cramps” and “hyperkinesia” in veterinary journals as well as online videos suggest that dystonic clinical signs deserve attention in veterinary practice. There is, however, little research on how closely these features resemble human dystonia or paroxysmal dyskinesias which are associated with dystonia. Since diagnosis of potential cases in domestic animals is important for prognosis, genetic counseling, and treatment of affected individuals, this review provides a comparative view on these movement disorders.

Academics of different expertise need to collaborate to understand the underlying pathogenesis of the dystonic syndrome (13, 14). The aim of this review is to explore the possibility that distinct neurological movement disorders in domestic animals, rarely described in the literature, represent a type of dystonia or dystonia-associated dyskinesia. As for laboratory animal models, much also could be learned from domestic animals about the pathogenesis of human dystonia (11). In the first part, we give a brief overview of the knowledge of human dystonia/paroxysmal dyskinesias and refer to several excellent reviews on this topic. In the second part, we draw comparisons between movement disorders in domestic animals and human dystonia/paroxysmal dyskinesias.

## Clinical and Neurophysiological Characteristics of Dystonia and Classification

Not all involuntary muscle contractions are dystonic in nature and there is no neurophysiological proof or confirmation of dystonia. However, there is a measurable neurophysiological defect in motor control, with excitability changes of interneural circuitry at spinal cord, brainstem (15, 16) and cortical level (17, 18), as well as the preparatory phase before voluntary movements (19, 20), which is possibly due to disturbed basal-ganglia nuclei input to these structures (21). A hallmark of dystonic contractions is their consistent directionality (22). The dystonic movements are patterned and repeatedly involve the same muscle groups within individuals. During dystonic movements, agonist and antagonist muscles typically contract simultaneously, thereby causing twisting of body parts. Poly-electromyographical recordings (poly-EMG: minimum of two channels) have shown three types of muscular activity during rest or voluntary movements: (1) long spasms, lasting several seconds, (2) repetitive rhythmical cocontracting spasms (200–500 ms), or (3) brief (<100 ms) irregular cocontracting jerks (23). Poly-EMG features of dystonia therefore match the clinical impression of cocontracting antagonistic muscles, with mainly tonic, non-reciprocal patterns during rest or voluntary movement (24–27). Dystonic movements often are longer lasting (or more persistent) than in other disorders such as myoclonus. Dystonia with myoclonic patterns are usually superimposed on long-lasting tonic activation (28). Dystonic spasms or jerky/myoclonic movements in dystonia clinically look similar to those of primary myoclonus but are not associated with electroencephalographic (EEG) changes (15). Repetitive contractions can cause jerking (tremulous) movements which might mimic tremor though can be distinguished by their irregular frequency. Dystonia is often worsened by voluntary movements or, as in cases of action or task-specific dystonia, present during voluntary movements and not during rest (e.g., writer’s cramp). Motor actions can cause dystonic symptoms in other parts of the body, called overflow phenomenon, for example writing can initiate leg dystonia (1). Fatigue and stress often cause aggravation, while the symptoms improve or disappear during relaxation and sleep (22).

Classification of dystonia has been modified extensively over the last few decades (1, 29). The current classification by clinical characteristics is summarized in Table 1. Classification by the age of onset is notable, because dystonia with first occurrence at an early age is more likely to progress from focal to generalized forms. For diagnosis and therapy, the classification by the affected body region and by the temporal pattern is important. Hemidystonia, for example, is often acquired. Task-specific and paroxysmal types have to be distinguished from pure action dystonia, where dystonic features accompany any voluntary movement. In paroxysmal dyskinesia, the same trigger might not always induce symptoms and the attacks last longer than the influence of the trigger. In contrast, action dystonia is predictably induced by the same voluntary motor activity and disappears when the inducing action is stopped (1). Paroxysmal dyskinesias can present with dystonia, choreic, ballistic, other, or a mixture of these hyperkinesias and can be inherited or acquired (30). According to the trigger, duration, frequency, and response to antiepileptic drugs, human paroxysmal dyskinesias are divided into subtypes (see Table 2). These paroxysmal dyskinesias often occur first in childhood or early adolescence (4).

### Differential Diagnoses

Twisting movements and postures can be caused by diverse disorders of the central and peripheral nervous systems or by non-neurological conditions. For example, tonic epileptic *seizures* can produce torsions. Those epileptic seizures are usually accompanied by a loss of consciousness, while dystonia (including paroxysmal types) occurs in awake individuals and in the absence of EEG changes (spikes and waves). Episodic dystonic features are not accompanied by autonomic signs and postictal behavior. Furthermore, an exaggerated startle reflex to tactile and acoustic stimuli, as known in *hyperekplexia* is not observed in dystonia. Awareness is not disturbed in dystonic patients, and there are no gross deficits in cognition (31). A hallmark of *spasticity* is the pathology of reflexes, while hyperreflexia is not typical of dystonia. However, moderate changes in spinal and brainstem reflexes like a decreased inhibition in blink reflex recovery can be present in patients with focal dystonia. Furthermore, transcranial magnetic stimulation (TMS) studies revealed a loss of inhibition of the primary motor cortex (32). *Myoclonus* differs concerning duration and electrophysiological characteristics, as described above. *Infections and peripheral alterations* have to be excluded, such as trochlear *nerve palsy* or vestibulopathy which can cause head tilt but not cervical dystonia. Sustained muscle contraction may have neuromuscular causes (e.g., neuromyotonia, *myotonic disorders*), and *tetanic spasms* can for example result from hypocalcemia (22). Not all motor dysfunctions in animals can be correlated to a specific human movement disorder. An example of this would be the inherited *hypertonicity syndrome* of Labrador Retrievers described by Vanhaesebrouck et al. (33), which is associated with a selective loss of spinal cord interneurons. As summarized in Table 3, different episodic neurological signs can occur in dogs and should be separated from paroxysmal dyskinesias.

**Table 3:** **Clinical characteristics of episodic disorders in animals**.

Discriminator	Paroxysmal dyskinesia	Idiopathic head tremor	Epileptic seizure	Vestibular attack	Syncope	Narcolepsy/Cataplexy	Neuromuscular weakness	Paroxysmal behavior changes (compulsive disorder)
Clinical status between episodes	Unremarkable	Unremarkable	Unremarkable or forebrain signs	Unremarkable	Normal heart rate or arrhythmia, pulse deficits, heart murmur, cyanosis, abnormal lung auscultation	Altered sleep/wake cycle, normal clinical examination	Unremarkable or generalized weakness, muscle atrophy, pain, and decreased reflexes	Unremarkable
Precipitating event or trigger	None or activity, exercise, excitement, and stress	None or stress, fatigue, and overstimulation	None or flashing lights, anxiety, and stress	None	Exercise, excitement, and stress	Excitement and eating	Activity and exercise	Behavioral triggers (e.g., fear)
Pre-event changes	Not applicable	Not applicable	Preictal signs may be observed including anxiety, restlessness, increased affection, contact-seeking, withdrawal, hiding, aggressiveness, and vocalization	Not applicable	Not applicable	Not applicable	Not applicable	Not applicable
Event description	Dystonia, chorea, ballismus, athetosis, tremors, impaired posture, and inability to stand or walk	Vertical or horizontal rhythmic head movement (5–8 Hz)	Depending on seizure focus, focal or generalized, tonic–clonic movements most common	Cardinal signs of vestibular dysfunction present: head tilt, nystagmus, vestibular ataxia, and collapse toward side of head tilt	Brief, sudden collapse, and rapid recovery	Sudden collapse	Stiff, stilted gait prior to collapse	Pacing, barking, licking, chasing imaginary objects or tail, and chewing objects
Level of consciousness	Unremarkable	Unremarkable	Often impaired or unconscious	Unremarkable or disorientated	Reduced to absent	Unremarkable if only cataplexy. Absent (asleep) in narcolepsy	Unremarkable	Unremarkable
Autonomic signs	Not applicable	Not applicable	Possible: hypersalivation, defaecation, and urination	Not applicable	Possible abnormalities of heart rate and rhythm	Not applicable	Not applicable	Not applicable
Muscle tone	Hypertonicity (focal or generalized)	Normal	Typically increased: tonic (hypertonicity) or alternating tonic–clonic movements	Unilateral decrease in extensor muscle tone	Flaccid (all body)	Flaccid (all body)	Often flaccid (can appear spastic with certain myopathies)	Normal
Lateralizing signs	Possible	Not applicable	Possible	Yes	Not applicable	Not applicable	Not applicable	Not applicable
Duration	Seconds to hours	Seconds to hours	Seconds to minutes or >5 min in case of status epilepticus	Seconds to hours	Seconds	Seconds to minutes	Minutes to hours	Minutes to hours
Postepisodic changes	Unremarkable or tiredness	Unremarkable, tiredness, or restlessness	Postictal signs frequently occur including disorientation, aggressive behavior, restlessness, pacing, lethargy, deep sleep, hunger, thirst, ataxia, proprioceptive deficits, and blindness	Not applicable	Not applicable	Not applicable	Not applicable	Not applicable
Further comments	Interaction with the owner can alleviate or interrupt the episode. Consider breed specific disorders and age at onset	Episodes can be interrupted in two thirds of the cases by the owner	Facial muscles often involved during the ictus	Subtle signs of vestibular disease might persist	Clinical signs of heart failure possible, e.g., dry cough and increased respiratory noise	Often occurs in young purebred dogs	May be accompanied by dysphagia, dysphonia, regurgitation, and dyspnea	History of anxiety disorder

In quadrupedal animals, dystonic signs are in part different to those in human patients. On four limbs, the animals have a better ability to compensate for moderate motor dysfunctions (11). Dystonic movements can present as gait abnormalities and dystonic postures of the body may result in a hunched or bent back. Severe ataxia, accompanied by muscle weakness, can lead to axial twisting movements which are not dystonic (34). The occurrence of muscle weakness always argues against the diagnosis of dystonia (35).

## Etiology and Pathophysiology

Dystonia was formerly divided into “primary (idiopathic) dystonia” and “secondary (symptomatic) dystonia.” Primary dystonia (as well as primary paroxysmal dyskinesias) was determined by the lack of a defined disease and the absence of neuropathological alterations, visible by brain imaging (MRI and CT) or by postmortem histological standard techniques (13). However, these examinations do not exclude changes in the density of specific types of neurons and microstructural alterations (11). Furthermore, there is a scarcity of human postmortal tissue donation suitable for more specific microscopic, neurochemical and molecular analyses (36).

With regard to new insights into the *etiology* of inherited dystonia, the division of primary versus secondary is no longer recommended (1). As extensively reviewed, gene mutations related to human dystonia currently designated as DYT1-25 (see Table 4) were isolated and for more than half of these genes encoded proteins were identified. The functions of these proteins include dopamine signaling, ion channels, protein chaperoning, transcriptional regulation, or transporters (37–40). A causative factor has been identified in around one-third of cases of human dystonia (1). As known for DYT1 (the most common inherited human dystonia), a gene defect does not always lead to a dystonic phenotype, i.e., the penetrance can be incomplete (2). Furthermore, a specific mutation can lead to various clinical phenotypes, indicating the importance of environmental or additional genetic factors in the pathophysiology. Alterations in neuronal plasticity, i.e., the ability of the nervous system to morphologically and/or biochemically change neural pathways and synapses in response to environmental factors, have been hypothesized to play an important role in dystonic signs (41–43). Thereby, various observations in humans and animal models can be explained (11), such as the overt neuronal dysfunction without apparent neuropathology and the often progressive nature.

**Table 4:** **Etiology of inherited dystonia in humans (38, 39, 37)**.

Designation	Clinical type	Clinical characteristics	Mode of inheritance	Gene locus	Gene
DYT1	Isolated dystonia	Early-onset generalized	Autosomal dominant	9q	*TOR1A*
DYT2	Isolated dystonia	Early-onset generalized	Autosomal recessive	Unknown	Unknown
DYT3	Combined, persistent dystonia	X-linked dystonia-parkinsonism; “lubag”	X-chromosomal recessive	Xq	*TAF1*
DYT4	Isolated dystonia	Whispering dysphonia	Autosomal dominant	19p	*TUBB4*
DYT5	Combined, persistent dystonia	Dopa-responsive dystonia; Segawa syndrome	Autosomal dominant	14q	*GCH1*
DYT6	Isolated dystonia	Adolescent-onset mixed phenotype	Autosomal dominant	8p	*THAP1*
DYT7	Isolated dystonia	Adult-onset focal	Autosomal dominant	18p	Unknown
DYT8	Combined, paroxysmal dystonia	Paroxysmal non-kinesigenic dyskinesia 1	Autosomal dominant	2q	*MR-1*
DYT10	Combined, paroxysmal dystonia	Paroxysmal kinesigenic dyskinesia 1	Autosomal dominant	16p-q	*PRRT2*
DYT11	Combined, persistent dystonia	Myoclonus-dystonia	Autosomal dominant	7q	*SGCE*
DYT12	Combined, persistent dystonia	Rapid-onset dystonia-parkinsonism	Autosomal dominant	19q	*ATP1A3*
DYT13	Isolated dystonia	Adolescent-onset multifocal/segmental	Autosomal dominant	1p	Unknown
DYT15	Combined, persistent dystonia	Myoclonus-dystonia	Autosomal dominant	18p	Unknown
DYT16	Combined, persistent dystonia	Early-onset generalized with parkinsonism	Autosomal recessive	2p	*PRKRA*
DYT17	Isolated dystonia	Adolescent-onset	Autosomal recessive	20pq	Unknown
DYT18	Combined, paroxysmal dystonia	Paroxysmal exertion-induced dyskinesia	Autosomal dominant	1p	*SLC2A1* (*GLUT1*)
DYT19	Combined, paroxysmal dystonia	Paroxysmal kinesigenic dyskinesia 2	Autosomal dominant	16q	Unknown
DYT20	Combined, paroxysmal dystonia	Paroxysmal non-kinesigenic dyskinesia 2	Autosomal dominant	2q	Unknown
DYT21	Isolated dystonia	Adult-onset generalized/multifocal	Autosomal dominant	2q	Unknown
DYT23	Isolated dystonia	Adult-onset cervical dystonia	Autosomal dominant	9q	*CIZ1*
DYT24	Isolated dystonia	Adult-onset craniocervical dystonia	Autosomal dominant	11p	*ANO3*
DYT25	Isolated dystonia	Adult-onset cervical dystonia	Autosomal dominant	18p	*GNAL*

Despite the varied etiology and often sporadic occurrence (no evidence for inheritance), there are probably common pathways in the *pathophysiology* of dystonia, though this is still poorly understood. There is strong evidence that dysfunction of the basal-ganglia nuclei and related thalamocortical network are essential (44, 45). Thus, symptomatic (acquired) dystonia is often related to brain lesions in the basal-ganglia and thalamic nuclei. Abnormal neuronal activity in the striatum, the input structure of the basal ganglia, can lead to disturbed inhibition of thalamocortical projections (11, 13, 46). Over time this may lead to an increase in striatal, cortical, and brain stem plasticity producing neural reorganization and symptoms of dystonia (4). This concept is in line with the observed improvement of dystonia after deep brain stimulations (DBS) of the globus pallidus internus (GPi), a basal-ganglia output structure. Although regarded as a basal-ganglia disorder, other brain regions can be involved in dystonic symptoms, such as the thalamus, cortex, brain stem, and the cerebellum (44, 47). Indeed, thalamocortical activities are controlled not only by the basal ganglia but also by the cerebellum. Cerebellar dysfunctions can be included [as indicated by clinical data and findings in animal models (10, 48–51)], although the most commonly recognized cerebellar signs ataxia, vestibular dysfunction, and ocular nystagmus are usually not present in dystonia. Functional neuroimaging data suggest that dystonia is a neurocircuit disorder, involving the cortico-striato-pallido-thalamo-cortical and cerebello-thalamo-cortical pathways (44).

Inherited, idiopathic types have been suggested to be related to neurochemical imbalances, including dopamine, acetylcholine, and GABAergic disinhibition (44, 46). Several lines of evidence indicated that striatal dopaminergic dysfunctions play an important role in the pathogenesis of dystonia (52, 53). Not all neurotransmitter systems have been well examined yet (e.g., serotonin and glutamate).

In summary, the etiology and pathophysiology of dystonic signs is heterogeneous. A very important research objective is the identification of common functional pathways to detect targets for therapeutics which are effective for a broader spectrum of different types of dystonia.

## Treatment

Except in some cases of acquired dystonia and inherited dopa-responsive dystonia in humans, causal or pathogenesis-targeted therapy is not available (54). Aims are to reduce or abolish involuntary movements and postures, relieve pain, and improve quality of life in human patients (55).

A licensed drug for symptomatic treatment of focal dystonias in humans is botulinum toxin (BoNT). It is the treatment of choice for focal dystonia, such as blepharospasm and cervical dystonia. After injections into dystonic muscles, the toxin blocks the release of acetylcholine for 2–4 months and thereby improves symptoms. Besides the modulation of motor output by blocking cholinergic synapses, it has been hypothesized that more sustained improvement results from modulating the central plasticity via the afferent system (56).

Empirical trials in human patients have shown that the response to systemic medication depends on the type of dystonia and shows individual diversity, as described by Jankovic (54, 55). Treatment with low doses of levodopa (precursor of dopamine) is very effective in patients with generalized dopa-responsive dystonia, which is caused by deficient dopamine synthesis. On the contrary, dopamine blocking or depleting agents, such as neuroleptics and tetrabenazine, sometimes improve other forms of dystonia. As known from trials with anticholinergic drugs, treatment over weeks with high doses is often needed before the severity of dystonic symptoms is reduced. Drugs which enhance GABAergic inhibition, such as baclofen (GABA_B_ receptor agonist) and benzodiazepines (diazepam and clonazepam) can also be effective. Specific types of paroxysmal dyskinesia improve by antiepileptic treatment (see Table 2).

Pharmacological treatment of generalized dystonia in humans is often ineffective. Myo- and neurotomy have been replaced by DBS, with the GPi as the target of choice (57, 58). Interestingly, patients often show a gradual and slow course of improvement, suggesting a role for secondary plastic changes in response to the changed basal-ganglia output from DBS (13).

## Dystonia in Lab Animal Models

During past decades, several animal models of dystonia have been proposed with the aim to give insights into its pathophysiology and to develop new therapeutics. It is well described that dystonic signs can be induced in animals or can be caused by spontaneous mutations in rodents. Based on the identification of gene defects in human dystonia/paroxysmal dyskinesia, mice and rats have been genetically modified and exhibit moderate motor disturbances. For detailed descriptions of animal models of dystonia, see previous reviews on this topic (9–12). Here, we wish to emphasize that spontaneous mutations leading to persistent generalized dystonic symptoms cause a low viability in affected rodents. Notably, detailed examinations (phenotyping) are important to decide if motor disturbances in laboratory animals actually represent dystonia. For example, dystonic episodes in *dt^sz^* mutant hamsters were initially misinterpreted as reflex epilepsy, although there is neither loss of consciousness nor EEG changes, autonomic signs or a response to antiepileptic drugs. Indeed, the signs in mutant hamsters show the characteristics of human paroxysmal non-kinesigenic dyskinesia with dystonia as the dominant feature and are based on basal-ganglia dysfunctions (59). Another example is the *Cacna1a* mutant tottering mouse which shows a persistent mild ataxia and brief epileptic seizures, but also episodic dyskinesia with coactivation of antagonistic muscles which are not accompanied by EEG changes. Dyskinesia in these mice is related to cerebellar dysfunction (5–10). In experimental animal models, EEG recordings can be done invasively via chronically implanted electrodes. In contrast, exclusion of spike-waves abnormalities by non-invasive EEG recordings in domestic animals during episodes is usually not possible because of muscle activity causing artifacts. This should be kept in mind regarding the descriptions of paroxysmal dyskinesias in dogs (see below).

## Hyperkinetic Movement Disorders in Domestic Animals: Common Features with Types of Human Dystonia and Dystonia-Associated Dyskinesias

The unspecific term “hyperkinesia” refers to different involuntary movements with increased motor activity. Several veterinary reports use this term to describe animals presenting dystonic-like features and paroxysmal dyskinesias. Most of these reports describe paroxysmal movement disorders (Table 5), difficult to differentiate from other episodic events, such as epileptic seizures (60). This does not mean that persistent types of dystonia do not occur in domestic animals; they may perhaps be under-reported in the literature if the degree of disability caused by the persistent generalized dystonia and/or an unsuccessful treatment lead to the decision to euthanize the affected animal. Severe types of dystonic postures can be painful in human patients (55), while this is unknown in affected animals. On the other hand, there may be mild forms or benign courses of dystonia in animals that do not require treatment (61) and/or are possibly not presented to a veterinarian.

**Table 5:** **Hyperkinetic disorders in domestic animals, possibly including dystonic signs**.

Species	Breed^a^	Characteristics indicating the presence of dystonia	Genetic defect/pathophysiology	Denomination in veterinary medicine	Assumed comparable type of human dystonia
**Dog**	Cavalier King Charles Spaniel	Episodes of involuntary twisting movements and abnormal postures of trunk and extremities with muscular hypertonicity, resulting in complete immobility	Autosomal-recessive mode of inheritance 15.7 kb deletion in the gene BCAN (encoding brain-specific extracellular matrix-proteoglycan brevican)	Episodic falling/hypertonicity/sudden collapse	Paroxysmal (non-)kinesiogenic dystonia
		Episodes are inducible by excitement/stress or exercise	
		Duration of episodes: up to minutes	
		Consciousness is unaltered	
		Treatment with benzodiazepines (clonazepam 0,5 mg/kg) and carbonic anhydrase inhibitors (acetazolamide) can be attempted	
	Scottish Terrier	Episodes of involuntary movements, starting with an increase in muscle tone and arching of the back, stiffness of extremities resulting in loss of balance	Unknown/autosomal-recessive mode of inheritance serotonergic and/or dopaminergic dysfunctions are presumed	Scottie cramp	Paroxysmal (non-)kinesiogenic dystonia
		Episodes are inducible by stress and effort/exercise as well as by amphetamine (induces dopaminergic overactivity)	
		Duration of episodes: up to minutes	
		Consciousness is unaltered	
		EMG abnormalities	
		Treatment with benzodiazepines (diazepam 0,5–1,5 mg/kg)	
	Chinook dog	Episodes of involuntary twisting movements and abnormal postures of trunk and extremities with muscular hypertonicity, partly with head tremor	Unknown/probably autosomal-recessive mode of inheritance	Chinook seizures	Paroxysmal dyskinesia
		Trigger: uncertain	
		Duration of episodes: up to 1 h	
		Consciousness is unaltered	
	Border Terrier	Episodes of involuntary movements	Unknown	Canine “epileptoid” cramping syndrome	Paroxysmal non-kinesiogenic dystonia
		Episodes are inducible by excitement and waking from sleep	
		Often associated with mild tremor and gastrointestinal signs (borborygmi, vomiting and diarrhea)	
		Duration of episodes: 2–30 min up to three times per day	
	Yorkshire Terrier	Episodes of generalized, sustained and painful involuntary muscle contractions	Unknown/case report no changes found by MRI and CT	–	Paroxysmal hyperkinetic disorder
		Duration of episodes up to 30 min, four times per day	
		Consciousness is unaltered	
	Boxer	Episodes of twisting movements and postures of trunk and extremities with muscle hypertonicity, torticollis, and “grimacing”	Unknown/hereditary	–	Paroxysmal dystonia
		Episodes are inducible by excitement	
		Duration of episodes: ~1–5 min	
		Consciousness is unaltered	
	Bichon Frisé	Episodes of abnormal twisting movements and postures of trunk and extremities, kyphosis, and “grimacing”	Unknown/case report	–	Paroxysmal hyperkinetic disorder
		Episodes occur spontaneously also at rest or are inducible by excitement/stress/effort	
		Consciousness is unaltered	
	German shorthaired pointer	Episodes of abnormal twisting movements and postures of trunk and extremities	Unknown/case report	–	Paroxysmal hyperkinetic disorder
		Episodes are inducible by stress/excitement/effort	
		Duration of episodes: 10 min to 3 h	
		Consciousness is unaltered	
		Successful treatment with phenobarbital (30 mg/kg)	
	Dalmatian dog	Hyperkinetic episodes with rigidity/hyperextension/twisting movements and postures of extremities as well as head and neck (torticollis), rigidity of paraspinal muscles, resulting in scoliosis and loss of balance/falling and immobility	Unknown/case report	–	Paroxysmal hyperkinetic disorder
		Episodes are inducible by excitement/stress and exercise	
		Duration of episodes: ~15–60 min	
		Consciousness is unaltered	
		Successful treatment with azetazolamide (0,1 mg/kg)	
	Great Dane	Bilateral blepharospasm	Unknown/case report	–	Focal dystonia (blepharospasm)
		Successful therapy with local application of botulinum toxin A	

**Horse**	Various breeds	Episodic occurrence of involuntary muscle contractions, mostly in the pelvic region and the hind limbs Episodes are inducible by exercise, stress and excitement Duration of episodes: seconds to minutes	Unknown/hereditary? End-terminal neuroaxonal lesion in the deep cerebellar nuclei	Shivering	Paroxysmal dystonia?
		Episodes of involuntary muscle contractions in the rear body parts but also in the nasolabial region	Unknown/	Stiff-horse syndrome	Paroxysmal dyskinesia?
		Episodes are inducible by exercise and excitement	

**Cattle**	Various breeds	Progressive consistent contractions of the hindlimb muscles	Unknown possibly autosomal recessive (reduced penetrance?)	Bovine spastic paresis	Limb dystonia
	Belgian Blue, Chianina, Dutch Improved Red and White	Stress/effort and external stimuli provoke enhanced muscle tone, resulting in hyperflexion of all extremities and disability to stand or walk	Mutation in the gene ATP2A1 (encoding for a Ca^2+-^ATPase [SERCA1] leads to disturbances in the Ca^2+^-reuptake from the cytosol in the endoplasmic reticulum)	Congenital muscular dystonia type 1 (CMD1)	Brody myopathy? (*not* dystonia)
	Belgian Blue	Acoustic or tactile stimuli are leading to episodes of sustained muscle contractions with twisting, repetitive movements and postures	Microdeletion in the gene Glyt2 (SLC6A5) (encoding for a presynaptic transporter of glycine)	Congenital muscular dystonia type 2 (CMD2)	Hyperekplexia/startle disease? (*not* dystonia)

*^a^For references, see text section*.

Descriptions of cases and postmortem brain examinations of euthanized animals could give interesting insights into the pathophysiology. The domestic dog offers unique advantages to identify gene defects and mechanisms of inherited diseases because of the sequenced canine genome and the closed population structures in the breeds. Therefore, canine models may provide a valuable tool for discovering disease alleles that have been difficult to find by studying human families or populations (61, 62).

### Motor Disturbances Which Possibly Share Common Features with Focal Dystonias

Focal dystonias involve a single body region, and may include involuntary rotation of the head/neck (cervical dystonia), dystonic muscle activation of the orbicularis oculi (blepharospasm), larynx (spasmodic dysphonia), or limb muscles (63). The initial symptoms can be mild and may be noticeable only after prolonged exertion or stress. The etiology is heterogeneous and multiple factors (e.g., overuse of muscles and central/peripheral insults) can be involved in their manifestation, probably related to loss of inhibition and disturbed sensorimotor integration (32, 64).

Focal dystonia can be experimentally induced in animals (65) and cases of focal muscle contractions in domestic animals may resemble human focal dystonia. Meyer-Lindenberg et al. (66) described an interesting case of severe bilateral *blepharospasm* in a Great Dane dog. Following a moderate bilateral ectropium, blepharospasm (which was intensified by stress and bright light) could be observed from an age of 8 months. The spasms worsened and resulted in a permanent closure of the eyelids. Topical anesthetic and systemic treatments, including tetrazepam, were not effective. EMG revealed complex high frequency discharges of the orbicularis oculi muscle. Finally, subcutaneous injections of BoNT in the area of the orbicularis oculi muscle resulted in disappearance of blepharospasm for 3–4 months (66). As observed in this dog, eye disorders commonly also precede human blepharospasm which is responsive to BoNT (63).

*Bovine spastic paresis* (*BoSP*) is a progressive neuromuscular disease which has been investigated for several decades, as recently reviewed by De Vlamynck et al. (67). BoSP occurs sporadically in most breeds of cattle with an estimated prevalence of 0.1–0.8. BoSP is currently considered to be inherited via autosomal recessive (not identified) gene(s) with incomplete penetrance. As in DYT1 dystonia in humans (13), it has been suggested that additional unidentified factors are important for the development of clinical signs in genetically predisposed calves (67). There are several obvious similarities to human limb dystonia. BoSP is commonly associated with repetitive contractions of the gastrocnemius muscles but also of other hindlimb muscles. Attempts to move are thought to aggravate cocontractions of extensors and flexors of the limb. The age of onset ranges from 1 day to 3 years. Starting with moderate symptoms, the clinical signs worsen over time and result in disabling unilateral or bilateral hyperextension of the hindlimb(s). EMG studies have demonstrated significantly increased activity of the gastrocnemius and superficial digital flexor muscles in standing animals. There is no medical treatment. Surgical procedures usually performed in affected calves include partial tibial neurectomy, but contractions of other muscles persist. It has been hypothesized that muscle contractions are caused by disinhibition of motor neurons. Pathomorphological changes were not detected (67). Interestingly, a lower concentration of homovanillic acid was found in the cerebrospinal fluid of affected calves, indicating that dopamine metabolism is reduced in the CNS (68). Recent cDNA microarray data, performed on spinal cords of BoSP and healthy calves of a Romagnola cattle breed pointed to a possible alteration of calcium signaling proteins (69). However, the etiology and pathogenesis of BoSP remain unknown.

### Generalized Movement Disorders Which Possibly Include Dystonic Features

#### Paroxysmal Dyskinesias in Dogs

*Episodic falling in Cavalier King Charles Spaniels* (*CKCS*), an autosomal-recessive inherited disorder (70, 71), shares similarities with human paroxysmal dyskinesia (61). In affected dogs, exercise and excitement can precipitate episodes of abnormal gait and fallings which last up to several minutes (Video S1 in Supplementary Material). There are no signs of an impairment of consciousness or autonomic dysfunction during or after these attacks (72). Affected animals develop an increased extensor muscle tone of all four limbs, resulting in an immobilized, “deer-stalking” or “praying” position, sometimes falling with legs held in extensor rigidity (70, 73). Other clinical signs are facial muscle stiffness, stumbling, a “bunny-hopping” gait, and arching of the back. It is unclear if cocontractions of flexors are involved. Initial clinical signs of the disease typically appear between 3 and 7 months of age. Interestingly, in some cases, there is spontaneous remission, as sometimes observed in human paroxysmal dyskinesia (74). There is no evidence for metabolic alterations as a cause, and light microscopic examinations have not revealed any lesions in the CNS or peripheral nerves (75). Clonazepam may be effective in suppressing the episodes in some cases (76). Recently, a 15.7 kb deletion in BCAN, encoding the brain-specific extracellular matrix-proteoglycan brevican, was found to be associated with episodic falling in CKCS (61, 77). This protein plays an important role in cell adhesion, migration, axon guidance, and neuronal plasticity (78). How this mutation leads to the manifestation of this hyperkinetic movement disorder in dogs has yet to be unraveled.

*Scottie cramp* is a paroxysmal hyperkinetic disorder in Scottish terriers (Video S2 in Supplementary Material), with onset usually occurring during the first year of life. This autosomal-recessive inherited disorder shows features of paroxysmal dyskinesia (72, 79–83). Affected dogs appear normal at rest. When performing lengthy exercise or excited, the dogs progressively develop a stiff, stilted gait over a few minutes. Initially, the dogs show an arching of the lumbar region of the back, progressing with a stiff-legged gait with the pelvic limbs overflexed and thoracic limbs abducted with increased extensor tone while walking. Occasionally, exaggerated flexion of the limbs against the body can be observed. The gait abnormalities progress until some dogs are unable to walk or fall into recumbency with a flexed neck and tail. Furthermore, clear abnormalities could be observed by EMG analyses during an episode, including higher amplitudes and longer duration of the interference pattern in affected muscles (84). There is a wide variety of clinical signs. While some Scottish terriers seem to rather exhibit myoclonic jerks, other dogs show a phenotype that clearly resemble dystonic movements [for video, see also Ref. (83)]. The affected dogs are fully conscious during the whole attacks, which usually last a few minutes. Affected dogs recover completely after a period of rest. No microscopically visible lesions have been found in the nervous system. The attacks of involuntary movements can be provoked by administration of amphetamine and can be suppressed with diazepam or acepromazine. In contrast, antiserotonin agents markedly increased the severity. While serotonin reuptake inhibitors can provoke acute dystonic symptoms in humans (85), fluoxetine and other drugs which enhance cerebral serotonin concentrations have been reported to reduce the severity of Scottie cramp (86). Therefore, it has been suggested that the syndrome may be related to a deficiency of serotonin, although quantification of brain serotonin content did not reveal differences from healthy dogs (87, 88). Severe forms of Scottie cramps are refractory to fluoxetine and show a better response to diazepam (83).

*Hereditary idiopathic paroxysmal dyskinesia in Chinook dogs* probably has an autosomal-recessive inheritance (89). The dogs exhibit episodes of dyskinesia with dystonia [e.g., torsions of limb(s)], chorea, and ballismus, and in some cases also head tremors. The attacks last from minutes to 1 h and can occur several times per day or less frequently (up to years between attacks). These episodes are not induced by sudden movements, and other triggers have not been described in Chinook dogs. Age of onset ranges from 2 months to 5 years, but usually the first attack appears during the first 3 years of life. The attacks are not associated with autonomic signs or loss of consciousness. EEG recordings demonstrated that the episodes usually occur in the absence of epileptic activity, but three dogs of one family exhibited generalized tonic–clonic epileptic seizures in addition to the clinical signs of paroxysmal dyskinesia. Indeed, it is known from human individuals that specific mutations can lead to a paroxysmal dyskinesia that is associated with epilepsy in some affected family members (90, 91). Therefore, Packer et al. (89) suggested that paroxysmal dyskinesia in Chinook dogs could result from a channelopathy, but further analyses are required to confirm this. Public videos show sequences of paroxysmal dyskinesia in Chinook dogs. The thorough description by Packer et al. (89) indicates that the movement disorder in the Chinook dogs shares common features with paroxysmal non-kinesigenic dyskinesia in human individuals.

*The canine* “*epileptoid*”*cramping syndrome* in the Border terrier (Video S3 in Supplementary Material) has been hypothesized to represent a form of paroxysmal non-kinesigenic dyskinesia (92). As indicated by a retrospective owner-directed study, the first episodes usually occur before 3 years of age. Although the episodes often appear spontaneously, owners identified excitement and waking from sleep as inducing factors. The majority of generalized attacks lasted 2–30 min, occurred up to three times per day and were often associated with mild tremor and borborygmi. Immediately before or after the episodes, gastrointestinal signs (vomiting and diarrhea) were observed in about 50% of the affected dogs, which has not been reported in human dystonia or paroxysmal dyskinesia. While antiepileptic drugs were ineffective, the movement disorder is responsive to a gluten-free diet. A recent study demonstrated a link of the condition to gluten sensitivity. Affected border terriers had increased serum titers of antitransglutaminase 2 and antigliadin antibodies which normalized when the dogs were fed a gluten-free diet (93).

*A movement disorder in boxer pups* shows similarities with features of human paroxysmal non-kinesigenic dyskinesia (94). Two unrelated litters of six (all affected) and nine (seven affected) pups developed “unusual neurological episodes,” triggered by excitement and lasting for 1–5 min, at 8 weeks of age. The frequency ranged from only two episodes in 6 months to 10 episodes per day. Interestingly, the frequency and duration of episodes decreased with age and were less frequent and less severe in females compared with males. Clinical signs included unilateral facial dystonia, producing grimacing, lifting of an extended thoracic limb which was either held elevated for several seconds or banged to the ground, unilateral dystonia of the neck and truncal muscles producing torticollis, and hyperflexion of a thoracic or pelvic limb. The pups were at all times conscious. MRI scans of the brain of an affected male pup did not show obvious abnormalities.

##### Case Reports of Episodic Movement Disorders in Dogs

As summarized in Table 5, there are further case reports of episodic hyperkinetic disorders in a Bichon frise (95), a German shorthaired pointer (96), and two Dalmatian dogs (97). Park et al. (98) described a paroxysmal movement disorder in a young Yorkshire terrier. The attacks of generalized, sustained muscle contractions, lasting about 30 min with a frequency of 4 per day, were accompanied neither by loss of consciousness nor by any alterations in the CNS, as determined by MRI and CT. Similar episodic movement disorders have been described in Springer Spaniel dogs, Norwich terrier, Soft Coated Wheaten terrier, and Maltese dogs (99–101), indicating that spontaneous or inherited paroxysmal dyskinesias occur in several dog breeds.

#### Generalized Clinical Signs in Horses

*Shivering*represents a neuromuscular syndrome in horses, recognized as early as 1910 (102). The horses exhibit episodes of involuntary muscle contractions with trembling of the pelvic limbs (shivering), sudden jerky extensor movements of the tail and raising of one hindlimb in a semiflexed, abducted position for a few seconds to minutes (103, 104). The horses show otherwise normal behavior. Shivering can occur at any age, and the severity may remain unchanged or may slowly progress (72). In more severe cases, involvement of the muscles of the forelimbs, the neck, the trunk, and the face is described (103). The involuntary movements are worsened by stress or excitement and can be abolished by rest. The cause of shivering is still unknown, and an effective treatment is not available. It has been suggested that shivering has a hereditary background and may involve neurochemical alterations in the basal-ganglia nuclei (103). However, recent immunohistochemical and ultrastructural investigations indicated that shivering is related to end-terminal neuroaxonal degeneration in the deep cerebellar nuclei (105). In view of some common symptoms, this neuromuscular syndrome in the horse may include dystonic features. Specific studies, such as EMG recordings, are necessary.

The *stiff-horse syndrome* is characterized by episodes of contractions in the muscles of the lower back and of the hindlimbs. Facial contractions may occur in some cases (106, 107). Usually, the episodes of muscle contractions, which last a few minutes, can be precipitated by stress but also by voluntary movements. EMG analysis showed persistent motor-unit activity over a few minutes in the axial and gluteal muscles (107). The stiff-horse syndrome has been presumed to be caused by a loss of GABAergic inhibition within the CNS (103). However, the cause is unknown, and it is unclear if this movement disorder in horses represents a type of paroxysmal dyskinesia or if it shares common features with the stiff-person-syndrome, characterized by progressive rigidity and muscle spasms affecting the axial and limb muscles (108).

Although this review focuses on hereditary dystonia/paroxysmal dyskinesias, we here refer to an interesting type of secondary dyskinesia with dystonic signs in horses. Chronic ingestions of yellow star thistle (*Centaurea solstitialis*) or Russian knapweed (*Centaurea repens*) can cause nigropallidal encephalomalacia in horses with an abrupt onset of neurologic signs, including the occurrence of dystonic symptoms in facial, lip, and tongue muscles as well as gait abnormalities (109). There is no loss of dopaminergic neurons, but lesions occur in the globus pallidus and in the substantia nigra pars reticularis (110), supporting the hypothesis that abnormal basal-ganglia output plays an important role in the pathophysiology.

#### Generalized Clinical Signs in Cattle

The so-called *congenital muscular dystonia* (CMD) type 1 and type 2 are reported to have an incidence of 0.1–0.2% in Belgian Blue cattle (111–113). All calves with CMD exhibit episodes of generalized muscles contractions with two distinct phenotypes. Calves with CMD1 show impaired swallowing, fatigue upon stimulation, and myotonia resulting in a complete stiffness of limbs. Usually, respiratory complications lead to death a few weeks after birth. CMD2 is characterized by severe episodes of myoclonus after acoustic or tactile stimuli (clinically resembling the disinhibited human startle reaction) with death of calves within a few hours after birth. These clinical signs obviously differ from human dystonic conditions. In fact, genetic analyses have revealed a muscle defect. CMD1 is associated with a mutation in the gene ATP2A1, encoding the fast-twitch skeletal muscle Ca^2+^-ATPase (SERCA1) that results in disturbed Ca^2+^ reuptake into the sarcoplasmatic reticulum at the end of the contractile phase (111). Therefore, CMD1 shows common mechanisms with Brody myopathy in humans (111) rather than with dystonia. Exercise-induced muscle contractions, termed as “pseudomyotonia” in Chianina cattle and in a Dutch cross-breed, are associated with SERCA1 gene (114–116). Furthermore, genetic analyses have shown that CMD2 is caused by a GlyT2 (SLC6A5) defect, comparable to the findings in human hyperekplexia and Irish Wolfhounds with hyperekplexia (111, 112, 117). Therefore, these movement disorders in cattle do not seem to be a type of dystonia and therefore should be renamed.

## Conclusion

Involuntary hyperkinetic movement disorders that share common clinical features with different types of dystonia and paroxysmal dyskinesias in humans are described in dogs, horses, and cattle. While genetic analyses have shown that the currently recognized movement disabilities termed as CMD in cattle do not seem to represent dystonia, it has yet to be clarified if hyperkinetic movements in dogs and horses are dystonic in nature. As neurophysiological examinations in patients with dystonia show specific EMG patterns, this may help to clarify diagnosis. Since muscle cocontractions of antagonistic muscles and phasic tremulous and/or non-tremulous muscle activity represent the hallmarks of dystonia, these would help to distinguish between possible diagnoses in affected animals. Although attempts at treatment with GABAmimetic drugs like benzodiazepines in dogs may be effective in some cases, the outcomes are inconsistent. Except for disorders with clearly defined genetic causes, the origin and the mechanisms underlying of the described movement disorders in domestic animals remain mostly unknown. Therefore, further postmortem studies and genetic analysis to determine the underlying pathophysiology and genetics of these conditions are required.

## Search Criteria

References for this review were identified through searches of PubMed up to September 2015, with the terms dystonia, dyskinesia, hyperkinesia, cramps, animals, dogs, cats, cattle, bovine, and horse. Only papers published in English were considered.

## Author Contributions

AR and MH wrote the first draft. JW and HV modified the final version.

## Conflict of Interest Statement

The authors declare that the research was conducted in the absence of any commercial or financial relationships that could be construed as a potential conflict of interest.

## References

[1] Albanese A Bhatia K Bressman SB Delong MR Fahn S Fung VS Phenomenology and classification of dystonia: a consensus update. Mov Disord (2013) 28:863–73. 10.1002/mds.25475 23649720 PMC3729880

[2] Rascol O Fabre N Bretel-Courbon C Ory-Magne F Perez-Lloret S Dyskinesias. In: Kompoliti K Verhagen L, editors. The Encyclopedia of Movement Disorders. New York, NY: Elsevier Academic Press (2010). p. 350–61.

[3] Richter A Sander SE Dyskinesias: Animal models. In: Kompoliti K Verhagen L, editors. The Encyclopedia of Movement Disorders. New York, NY: Elsevier Academic Press (2010). p. 361–5.

[4] Jankovic J Demirkiran M Classification of paroxysmal dyskinesias and ataxias. Adv Neurol (2002) 89:387–400. 11968463

[5] Sanger TD Chen D Fehlings DL Hallett M Lang AE Mink JW Definition and classification of hyperkinetic movements in childhood. Mov Disord (2010) 25(11):1538–49. 10.1002/mds.23088 20589866 PMC2929378

[6] Elia AE Lalli S Albanese A. Differential diagnosis of dystonia. Eur J Neurol (2010) 17(Suppl 1):1–8. 10.1111/j.1468-1331.2010.03052.x 20590801

[7] Defazio G. The epidemiology of primary dystonia: current evidence and perspectives. Eur J Neurol (2010) 17(Suppl 1):9–14. 10.1111/j.1468-1331.2010.03053.x 20590802

[8] Jinnah HA Hess EJ. Experimental therapeutics for dystonia. Neurotherapeutics (2008) 5:198–209. 10.1016/j.nurt.2008.01.001 18394563 PMC2322876

[9] Jinnah H Richter A Mink JW Caldwell GA Caldwell KA Gonzalez-Alegre P Animal models for drug discovery in dystonia. Expert Opin Drug Discov (2008) 3:83–97. 10.1517/17460441.3.1.83 23480141

[10] Richter A Löscher W Pathophysiology of idiopathic dystonia: findings from genetic animal models. Prog Neurobiol (1998) 54:633–77. 10.1016/S0301-0082(97)00089-0 9560845

[11] Richter F Richter A. Genetic animal models of dystonia: common features and diversities. Prog Neurobiol (2014) 121C:91–113. 10.1016/j.pneurobio.2014.07.002 25034123

[12] Wilson BK Hess EJ. Animal models for dystonia. Mov Disord (2013) 28:982–9. 10.1002/mds.25526 23893454 PMC3728703

[13] Breakefield XO Blood AJ Li Y Hallett M Hanson PI Standaert DG. The pathophysiological basis of dystonias. Nat Rev Neurosci (2008) 9:222–34. 10.1038/nrn2337 18285800

[14] Richter A Richter F What can we learn from animal models of dystonia/dyskinessias in veterinary and human neurology? Proceedings 28th ESVN-ECVN Congress; Amsterdam (2015). 35 p. Available from: http://veterinair-neuroloog.nl/content/2-onderzoek/3-movement-disorders/proceedings-movement-disorders-congres-2015

[15] Rothwell JC Obeso JA Day BL Marsden CD Pathophysiology of dystonias. motor control mechanisms in health and disease. In: Desmedt JE, editor. Motor Control Mechanisms in Health and Disease. New York, NY: Raven Press (1983). p. 851–72.

[16] Tolosa E Montserrat L Bayes A. Blink reflex studies in focal dystonias: enhanced excitability of brainstem interneurons in cranial dystonia and spasmodic torticollis. Mov Disord (1988) 3:61–9. 10.1002/mds.870030108 3173365

[17] Ikoma K Samii A Mercuri B Wassermann EM Hallett M. Abnormal cortical motor excitability in dystonia. Neurology (1996) 46:1371–6. 10.1212/WNL.46.5.1371 8628484

[18] Ridding MC Sheean G Rothwell JC Inzelberg R Kujirai T. Changes in the balance between motor cortical excitation and inhibition in focal, task specific dystonia. J Neurol Neurosurg Psychiatry (1995) 59:493–8. 10.1136/jnnp.59.5.493 8530933 PMC1073711

[19] Deuschl G Toro C Matsumoto J Hallett M Movement-related cortical potentials in writer’s cramp. Ann Neurol (1995) 38:509–14. 10.1002/ana.410380606 8526458

[20] Kaji R Ikeda A Ikeda T Kubori T Mezaki T Kohara N Physiological study of cervical dysonia. Task specific abnormality in contingent negative variation. Brain (1995) 118(Pt 2):511–22. 10.1093/brain/118.2.511 7735891

[21] Byrnes ML Thickbroom GW Wilson SA Sacco P Shipman JM Stell R The corticomotor representation of upper limb muscles in writer’s cramp and changes following botulinum toxin injection. Brain (1998) 121(Pt 5):977–88. 10.1093/brain/121.5.977 9619198

[22] Geyer HI Bressman SB. The diagnosis of dystonia. Lancet Neurol (2006) 5:780–90. 10.1016/S1474-4422(06)70547-6 16914406

[23] Rothwell JC The physiology of dystonia. In: Tsui JKC Calne DB, editors. Handbook of Dystonia. New York, NY: Dekker (1995). p. 59–76.

[24] Berardelli A Rothwell JC Hallett M Thompson PD Manfredi M Marsden CD. The pathophysiology of primary dystonia. Brain (1998) 121(Pt 7):1195–212. 10.1093/brain/121.7.1195 9679773

[25] Deuschl G Heinen F Kleedorfer B Wagner M Lücking CH Poewe W. Clinical and polymyographic investigation of spasmodic torticollis. J Neurol (1992) 1:9–16. 10.1007/BF00839204 1541974

[26] Wissel J Schelosky L Ebersbach G Poewe W The concept of ‘dystonic tremor’. Mov Disord (1992) 7(Suppl 1):90.1557074

[27] Yanagisawa N Goto A Dystonia musculorum deformans: analysis with electromyography. J Neurol Sci (1971) 13:39–65. 10.1016/0022-510X(71)90206-1 5566110

[28] Obeso JA Rothwell JC Day BL Marsden CD. Myoclonic dystonia. Neurology (1983) 33:825–30. 10.1212/WNL.33.7.825 6683367

[29] Fahn S Classification of movement disorders. Mov Disord (2011) 26:947–57. 10.1002/mds.23759 21626541

[30] Erro R Sheerin UM Bhatia KP. Paroxysmal dyskinesias revisited: a review of 500 genetically proven cases and a new classification. Mov Disord (2014) 29:1008–16. 10.1002/mds.25933 24963779

[31] Kuyper DJ Parra V Aerts S Okun MS Kluger BM. Nonmotor manifestations of dystonia: a systematic review. Mov Disord (2011) 26:1206–17. 10.1002/mds.23709 21484874 PMC3652664

[32] Hallett M. Neurophysiology of dystonia: the role of inhibition. Neurobiol Dis (2011) 42:177–84. 10.1016/j.nbd.2010.08.025 20817092 PMC3016461

[33] Vanhaesebrouck AE Shelton GD Garosi L Harcourt-Brown TR Couturier J Behr S A novel movement disorder in related male Labrador Retrievers characterized by extreme generalized muscular stiffness. J Vet Intern Med (2011) 25:1089–96. 10.1111/j.1939-1676.2011.0757.x 21781161

[34] Hamann M Meisler M Richter A. Motor disturbances in mice with deficiency of the sodium channel gene Scn8a show features of human dystonia. Exp Neurol (2003) 184:830–8. 10.1016/S0014-4886(03)00290-5 14769375

[35] Malone A Manto M Hass C. Dissecting the links between cerebellum and dystonia. Cerebellum (2014) 13:666–8. 10.1007/s12311-014-0601-4 25239288

[36] Paudel R Hardy J Revesz T Holton JL Houlden H. Review: genetics and neuropathology of primary pure dystonia. Neuropathol Appl Neurobiol (2012) 38:520–34. 10.1111/j.1365-2990.2012.01298.x 22897341

[37] Klein C Genetics in dystonia. Parkinsonism Relat Disord (2014) 20(Suppl 1):137–42. 10.1016/S1353-8020(13)70033-6 24262166

[38] Lohmann K Klein C. Genetics of dystonia: what’s known? What’s new? What’s next? Mov Disord (2013) 28:899–905. 10.1002/mds.25536 23893446

[39] Müller U. The monogenic primary dystonias. Brain (2009) 132:2005–25. 10.1093/brain/awp172 19578124

[40] Ozelius LJ Lubarr N Bressman SB. Milestones in dystonia. Mov Disord (2011) 26:1106–26. 10.1002/mds.23775 21626555

[41] Peterson DA Sejnowski TJ Poizner H. Convergent evidence for abnormal striatal synaptic plasticity in dystonia. Neurobiol Dis (2010) 37:558–73. 10.1016/j.nbd.2009.12.003 20005952 PMC2846420

[42] Quartarone A Hallett M. Emerging concepts in the physiological basis of dystonia. Mov Disord (2013) 28:958–67. 10.1002/mds.25532 23893452 PMC4159671

[43] Quartarone A Pisani A. Abnormal plasticity in dystonia: Disruption of synaptic homeostasis. Neurobiol Dis (2011) 42:162–70. 10.1016/j.nbd.2010.12.011 21168494

[44] Alongi P Laccarino L Perani D. PET neuroimaging: insights on dystonia and tourette syndrome and potential applications. Front Neurol (2014) 5:183. 10.3389/fneur.2014.00183 25295029 PMC4171987

[45] Stoessl AJ Lehericy S Strafella AP Imaging insights into basal ganglia function, Parkinson’s disease, and dystonia. Lancet (2014) 384:532–44. 10.1016/S0140-6736(14)60041-6 24954673 PMC4454525

[46] Gittis AH Kreitzer AC. Striatal microcircuitry and movement disorders. Trends Neurosci (2012) 35:557–64. 10.1016/j.tins.2012.06.008 22858522 PMC3432144

[47] Wichmann T Dostrovsky JO Pathological basal ganglia activity in movement. Neuroscience (2011) 198:232–44. 10.1016/j.neuroscience.2011.06.048 21723919 PMC3209553

[48] Argyelan M Carbon M Niethammer M Ulug AM Voss HU Bressman SB Cerebellothalamocortical connectivity regulates penetrance in dystonia. J Neurosci (2009) 29:9740–7. 10.1523/JNEUROSCI.2300-09.2009 19657027 PMC2745646

[49] Fremont R Khodakhah K Alternative approaches to modeling hereditary dsytonias. Neurotherapeutics (2012) 9(2):315–22. 10.1007/s13311-012-0113-1 22422472 PMC3337017

[50] Prudente CN Hess EJ Jinnah HA. Dystonia as a network disorder: what is the role of the cerebellum? Neuroscience (2014) 260:23–35. 10.1016/j.neuroscience.2013.11.062 24333801 PMC3928686

[51] Raike RS Jinnah HA Hess EJ. Animal models of generalized dystonia. NeuroRx (2005) 2:504–12. 10.1602/neurorx.2.3.504 16389314 PMC1144494

[52] Bragg DC Armata IA Nery FC Breakefield XO Sharma N. Molecular pathways in dystonia. Neurobiol Dis (2011) 42:136–47. 10.1016/j.nbd.2010.11.015 21134457 PMC3073693

[53] Goodchild RE Grundmann K Pisani A. New genetic insights highlight ‘old’ ideas on motor dysfunction in dystonia. Trends Neurosci (2013) 36:717–25. 10.1016/j.tins.2013.09.003 24144882

[54] Jankovic J. Medical treatment of dystonia. Mov Disord (2013) 28:1001–12. 10.1002/mds.25552 23893456

[55] Jankovic J. Treatment of hyperkinetic movement disorders. Lancet Neurol (2009) 8:844–56. 10.1016/S1474-4422(09)70183-8 19679276

[56] Gilio F Curra A Lorenzano C Modugno N Manfredi M Berardelli A. Effects of botulinum toxin type A on intracortical inhibition in patients with dystonia. Ann Neurol (2000) 48(1):20–6. 10.1002/1531-8249(200007)48:1<20::AID-ANA5>3.3.CO;2-L 10894212

[57] Thenganatt MA Jankovic J. Treatment of dystonia. Neurotherapeutics (2014) 11:139–52. 10.1007/s13311-013-0231-4 24142590 PMC3899473

[58] Brüggemann N Kühn A Schneider SA Kamm C Wolters A Krause P Short- and long-term outcome of chronic pallidal neurostimulation in monogenic isolated dystonia. Neurology (2015) 84(9):895–903. 10.1212/WNL.0000000000001312 25653290 PMC6170184

[59] Richter A The genetically dystonic hamster: an animal model of paroxysmal dystonia. In: LeDoux M, editor. Animal Models of Movement Disorders. New York, NY: Elsevier Academic Press (2005). p. 459–66.

[60] Packer RM Berendt M Bhatti S Charalambous M Cizinauskas S De Risio L Inter-observer agreement of canine and feline paroxysmal event semiology and classification by veterinary neurology specialists and non-specialists. BMC Vet Res (2015) 11(1):39. 10.1186/s12917-015-0356-2 25881213 PMC4337258

[61] Forman OP Penderis J Hartley C Hayward LJ Ricketts SL Mellersh CS. Parallel mapping and simultaneous sequencing reveals deletions in BCAN and FAM83H associated with discrete inherited disorders in a domestic dog breed. PLoS Genet (2012) 8(1). 10.1371/journal.pgen.1002462 22253609 PMC3257292

[62] Ostrander EA Beale HC. Leading the way: finding genes for neurologic disease in dogs using genome-wide mRNA sequencing. BMC Genet (2012) 13:56. 10.1186/1471-2156-13-56 22781504 PMC3409065

[63] Jinnah HA Berardelli A Comella C Defazio G Delong MR Factor S The focal dystonias: current views and challenges for future research. Mov Disord (2013) 28:926–43. 10.1002/mds.25567 23893450 PMC3733486

[64] Altenmüller E Jabusch HC. Focal dystonia in musicians: phenomenology, pathophysiology and triggering factors. Eur J Neurol (2010) 17(Suppl 1):31–6. 10.1111/j.1468-1331.2010.03048.x 20590806

[65] Evinger C. Animal models of focal dystonia. NeuroRx (2005) 2:513–24. 10.1602/neurorx.2.3.513 16389315 PMC1144495

[66] Meyer-Lindenberg A Wohlfarth KM Switzer EN. The use of botulinum toxin A for treatment of possible essential blepharospasm in a dog. Aust Vet J (2003) 81(19):612–4. 10.1111/j.1751-0813.2003.tb12503.x 15080472

[67] De Vlamynck C Pille F Vlaminck L. Bovine spastic paresis: current knowledge and scientific voids. Vet J (2014) 202(2):229–35. 10.1016/j.tvjl.2014.07.015 25201252

[68] De Ley G De Moor A Bovine spastic paralysis: cerebrospinal fluid concentrations of homovanillic acid and 5-hydrosyindoleacetic acid in normal and spastic calves. American J Vet Res (1975) 36:227–8. 1111389

[69] Pariset L Bongiorni S Bueno S Gruber CEM Prosperini G Chillemi G Microarray gene expression profiling of neural tissues in bovine spastic paresis. BMC Vet Res (2013) 9:122. 10.1186/1746-6148-9-122 23782433 PMC3693873

[70] Herrtage ME Palmer AC Episodic falling in the cavalier King Charles spaniel. Vet Rec (1983) 112:458–9. 10.1136/vr.112.19.458 6868317

[71] Rusbridge C. Neurological diseases of the Cavalier King Charles Spaniel. J Small Anim Pract (2005) 46(6):265–72. 10.1111/j.1748-5827.2005.tb00319.x 15971896

[72] De Lahunta A Glass E Upper motor neuron: movement disorders. In: De Lahunta A Glass E, editors. Veterinary Neuroanatomy and Clinical Neurology. St. Louis, MO: Saunders/Elsevier (2009). p. 217–20.

[73] Wright JA Brownlie SE Smyth JB Jones DG Wotton P Muscle hypertonicity in the cavalier King Charles spaniel–myopathic features. Vet Rec (1986) 118(18):511–2. 10.1136/vr.118.18.511 3716135

[74] Nardocci N Fernandez-Alvarez E Wood NW Spacy SD Richter A The paroxysmal dyskinesias. In: Guerrini R Aicardi J Andermann F Hallett M, editors. Epilepsy and Movement Disorders. Cambridge: Cambridge University Press (2002). p. 125–39.

[75] Wright JA Smyth JBA Brownlie SE Robins M. A myopathy associated with muscle hypertonicity in the Cavalier King Charles Spaniel. J Comp Pathol (1987) 97(5):559–65. 10.1016/0021-9975(87)90006-5 3680644

[76] Garosi LS Platt SR Shelton GD Hypertonicity in Cavalier King Charles spaniels. J Vet Intern Med (2002) 16:330.

[77] Gill JL Tsai KL Krey C Noorai RE Vanbellinghen JF Garosi LS A canine BCAN microdeletion associated with episodic falling syndrome. Neurobiol Dis (2012) 45:130–6. 10.1016/j.nbd.2011.07.014 21821125 PMC3898273

[78] Brakebusch C Seidenbecher CI Asztely F Rauch U Matthies H Meyer H Brevican-deficient mice display impaired hippocampal CA1 long-term potentiation but show no obvious deficits in learning and memory. Mol Cell Biol (2002) 22:7417–27. 10.1128/MCB.22.21.7417-7427.2002 12370289 PMC135663

[79] Clemmons RM Peters RI Meyers KM Scotty cramp: a review of cause, characteristics, diagnosis, and treatment. Compendium Contin Educ Pract Vet (1980) 2:385.

[80] Garosi L Harvey RJ Scottie cramp and canine epileptoid cramping syndrome in Border terriers. Vet Rec (2012) 170:186–7. 10.1136/vr.e1127 22354655

[81] Meyers KM Lund JE Padgett GA Hyperkinetic episodes in Scottish terrier dogs. J Am Vet Med Assoc (1969) 155:129. 5816228

[82] Meyers KM Padgett GA Dickson WM The genetic background of a kinetic disorder of Scottish terrier dogs. J Hered (1970) 61:189–92. 5519667 10.1093/oxfordjournals.jhered.a108080

[83] Urkasemsin G Olby NJ Clinical characteristics of Scottie Cramp in 31 cases. J Small Anim Pract (2015) 56:276–80. 10.1111/jsap.12317 25599802

[84] Meyers KM Dickson WM Lund JE Padgett GA Muscular hypertonicity. Episodes in Scottish terrier dogs. Arch Neurol (1971) 25:61–8. 10.1001/archneur.1971.00490010071010 5169523

[85] Burkhard PR. Acute and subacute drug-induced movement disorders. Parkinsonism Relat Disord (2014) 20(Suppl 1):S108–12. 10.1016/S1353-8020(13)70027-0 24262159

[86] Geiger KM Klopp LS. Use of a selective serotonin reuptake inhibitor for treatment of episodes of hypertonia and kyphosis in a young adult Scottish Terrier. J Am Vet Med Assoc (2009) 235(2):168–71. 10.2460/javma.235.2.168 19601736

[87] Meyers KM Dickson WM Schaub RG Serotonin involvement in a motor disorder of Scottish terrier dogs. Life Sci (1973) 13:1261–74. 10.1016/0024-3205(73)90011-8 4847792

[88] Peters RI Meyers KM Precursor regulation of serotonergic neuronal function in Scottish Terrier dogs. J Neurochem (1977) 29(4):753–5. 10.1111/j.1471-4159.1977.tb07796.x 591951

[89] Packer RA Patterson EE Taylor JF Coates JR Schnabel RD O’Brien DP. Characterization and mode of inheritance of a paroxysmal dyskinesia in Chinook dogs. J Vet Intern Med (2010) 24:1305–13. 10.1111/j.1939-1676.2010.0629.x 21054538

[90] Du W Bautista JF Yang H Diez-Sampedro A You SA Wang L Calcium-sensitive potassium channelopathy in human epilepsy and paroxysmal movement disorder. Nat Genet (2005) 37:733–8. 10.1038/ng1585 15937479

[91] Suls A Dedeken P Goffin K Van Esch H Dupont P Cassiman D Paroxysmal exercise-induced dyskinesia and epilepsy is due to mutations in SCL2A1, encoding the glucose transporter GLUT1. Brain (2008) 131:1831–44. 10.1093/brain/awn113 18577546 PMC2442425

[92] Black V Garosi L Lowrie M Harvey RJ Gale J. Phenotypic characterisation of canine epileptoid cramping syndrome in the Border terrier. J Small Anim Pract (2014) 55:102–7. 10.1111/jsap.12170 24372194 PMC4277704

[93] Lowrie M Garden O Hadjivassiliou M Harvey R Sanders D Powell R Canine epileptoid cramping syndrome: a gluten-sensitive paroxysmal movement disorder – more than a gut feeling. Proceedings 28th ESVN-ECVN Congress; Amsterdam (2015). 42 p. Available from: http://veterinair-neuroloog.nl/content/2-onderzoek/3-movement-disorders/proceedings-movement-disorders-congres-2015

[94] Ramsey IK Chandler KE Franklin RJM A movement disorder in boxer pups. Vet Rec (1999) 144(7):179–80. 10.1136/vr.144.7.179 10097327

[95] Penderis J Franklin RJ. Dyskinesia in an adult bichon frise. J Small Animal Pract (2001) 42:24–5. 10.1111/j.1748-5827.2001.tb01979.x 11219819

[96] Harcourt-Brown T. Anticonvulsant responsive, episodic movement disorder in a German shorthaired pointer. J Small Anim Pract (2008) 49:405–7. 10.1111/j.1748-5827.2008.00540.x 18631228

[97] Woods CB Hyperkinetic episodes in two Dalmatian dogs. J Am Anim Hosp Assoc (1977) 13:255–7.

[98] Park HJ Seo DK Song KH Seo KW. Paroxysmal dyskinesia suspected as canine epileptoid cramping syndrome in a young Yorkshire terrier dog. J Vet Med Sci (2014) 76:1129–32. 10.1292/jvms.14-0015 24805907 PMC4155193

[99] Di Riso L Freeman J Epileptoid cramping syndrome in the Norwich terrier: clinical characterization and prevalence in the UK. Proceedings 28th ESVN-ECVN Congress; Amsterdam (2015). 38 p. Available from: http://veterinair-neuroloog.nl/content/2-onderzoek/3-movement-disorders/proceedings-movement-disorders-congres-2015

[100] Martlé V Bhatti S O’Brien D Gielen I Van Ham L Paroxysmal dyskinesia in adult Maltese dogs? Proceedings 28th ESVN-ECVN Congress; Amsterdam (2015). p. 62–3. Available from: http://veterinair-neuroloog.nl/content/2-onderzoek/3-movement-disorders/proceedings-movement-disorders-congres-2015

[101] Shelton GD Muscle pain, cramps and hypertonicity. Vet Clin North Am Small Anim Pract (2004) 34(6):1483–96. 10.1016/j.cvsm.2004.05.019 15474685

[102] McCall JR “Stringhalt” and “shivering”. Proceedings 28th General Meeting of the National Veterinary Association (1910). p. 23–56.

[103] Baird JD Firshman AM Valberg SJ Shivers (Shivering) in the horse: a review. AAEP Proc (2006) 52:359–64.

[104] Draper AC Bender JB Firshman AM Baird JD Reed S Mayhew IG Epidemiology of shivering (shivers) in horses. Equine Vet J (2015) 47(2):182–7. 10.1111/evj.12296 24802303

[105] Valberg SJ Lewis SS Shivers JL Barnes NE Konczak J Draper AC The equine movement disorder “Shivers” is associated with selective cerebellar purkinje cell axonal degeneration. Vet Pathol (2015). 10.1177/0300985815571668 25714471

[106] Nollet H Vanderstraeten G Sustronck B Van Ham L Ziegler M Deprez P Suspected case of stiff-horse syndrome. Vet Rec (2000) 146:282–4. 10.1136/vr.146.10.282 10749042

[107] Nollet H Van Ham L Vanderstraeten G Deprez P Stiff Horse syndrome? J Vet Am Int Med (2005) 19:288. 10.1136/vr.146.10.282 10749042

[108] Baizabal-Carvallo JF Jankovic J. Stiff-person syndrome: insights into a complex autoimmune disorder. J Neurol Neurosurg Psychiatry (2015) 86(8):840–8. 10.1136/jnnp-2014-309201 25511790

[109] Chang HT Rumbeiha WK Patterson JS Puschner B Knight A Goudreau J Revisiting the neuropathology of equine nigropallidal encephalomalacia: a novel animal model for environmental etiology of Parkinson disease? Proceedings of the Annual Meeting of the Society for Neuroscience; Washington, DC (2008).

[110] Chang HT Rumbeiha WK Patterson JS Puschner B Knight AP. Toxic equine parkinsonism: an immunohistochemical study of 10 horses with nigropallidal encephalomalacia. Vet Pathol (2012) 49:398–402. 10.1177/0300985811406885 21527781

[111] Charlier C Coppieters W Rollin F Desmecht D Agerholm JS Cambisano N Highly effective SNP-based association mapping and management of recessive defects in livestock. Nat Genet (2008) 40:449–54. 10.1038/ng.96 18344998

[112] Gill JL James VM Carta E Harris D Topf M Scholes SFE Identification of congenital muscular dystonia 2 associated with an inherited GlyT2 defect in Belgian Blue cattle from the United Kingdom. Animal Genet (2012) 43:267–70. 10.1111/j.1365-2052.2011.02255.x 22486497

[113] Harvey RJ Topf M Harvey K Rees MI. The genetics of hyperekplexia: more than startle! Trends Genet (2008) 24:439–47. 10.1016/j.tig.2008.06.005 18707791

[114] Grünberg W Sacchetto R Wijnberg I Neijenhuis K Mascarello F Damiani E Pseudomyotonia, a muscle function disorder associated with an inherited ATP2A1 (SERCA1) defect in a Dutch Improved Red and White cross-breed calf. Neuromusc Disord (2010) 20:467–70. 10.1016/j.nmd.2010.04.010 20547455

[115] Testoni S Boni P Gentile A Congenital pseudomyotonia in Chianina cattle. Vet Rec (2008) 163:252 10.1136/vr.163.8.252 18723868

[116] Sacchetto R Testoni S Gentile A Damiani E Rossi M Liguori R A defective SERCA1 protein is responsible for congenital pseudomyotonia in Chianina cattle. Am J Pathol (2009) 174(2):565–73. 10.2353/ajpath.2009.080659 19116366 PMC2630564

[117] Gill JL Capper D Vanbellinghen JF Chung SK Higgins RJ Rees MI Startle disease in Irish wolfhounds associated with a microdeletion in the glycine transporter GlyT2 gene. Neurobiol Dis (2011) 43(1):184–9. 10.1016/j.nbd.2011.03.010 21420493 PMC4068303

